# 

**Published:** 1880-05-20

**Authors:** 


### TABLE OF CONTENTS.

ORIGINAL AND SELECTED ARTICLES.

Some Objections to the Present Theories Regarding Yellow Fever, by T. B. Greenley, of Kentucky, 153. Cheloid, by Lindsay Johnson, M.D , 158. Is it Inherited Paralysis? by L. W. Weathers, M.D.; Partial Paralysis and want of co-ordination from irritation of the Genital Organs, by Lewis A. Sayre. M.D., 160. Insanity a symptom of Uterine Disease, 164. New Method of Arresting Post Partum Hemorrhage, 166.

ABSTRACTS AND GLEANINGS.

Sims' Speculum always at Hand, 169. Ringworm of the Scalp, 170. Painless Cure of Internal Hemorrhoids, 171. Pneumonitis, 174. Resuscitation of a Still Born Infant nearly an Hour after Birth; Gelsemium in Facial Neuralgia, 175. Suction Operation for Cataract, 176. Tarsal Ophthalmia; Skin Grafting, 177. Iridin and Enonymin on Man; New Dressing for Wounds, 178. Don't Blow your Nose; Venesection; Bismuth Snuff, 179. Rubeola in a Pregnant and Puerperal Woman; Oleomargarin as a Food Product; Sweet Cassava Root, 180. Guarana; Antidote to Arsenic; Pruritus Ani; Suppression of Menses, 181. Dover's Powder for Night-Sweats of Phthisis; Balsam of Peru in Pruritus; Incubation Period of Scarlatina; About Anæsthetics; Improved Nitrate of Siver Caustic, 182.

SCIENTIFIC ITEMS.

Audiphone and Dentaphone; Capital Punishment by Electricity; Power of Imagination, 183. The Topophone; A Mechanical Watch-Dog; The Winter of 1879-1880; The Metric System, 184.

PRACTICAL NOTES AND FORMULÆ.

Hemorrhgic Malarial Fever; Hay Fever; Gonorrhœa: For Irregular Menstruation; Cubebs in Whooping Cough, 185. Compound Solution of Carbolic Acid: Damiana, 186. Senecio Aureus in Rheumatism; Excipient for Quinia Pills; In Menopause; Antidote for Carbolic Acid, 187. Compound Wine of Iron and Calisaya; Spirit of Nitrous Ether; Pruritus Ani; For After-Pains, 188.

EDITORIAL AND MISCELLANEOUS.

Please Read; Editorial Notices, 189. American Medical Association; McDowell Medical Society; Medical Association of Georgia, 190. Hydrate of Chloral; Special Notices, 192.

